# The establishment and optimization of a *Mycoplasma pneumoniae* detection system based on ERA-CRISPR/Cas12a

**DOI:** 10.1128/spectrum.03235-24

**Published:** 2025-02-25

**Authors:** Fo Yang, Qianlin Wu, Xiaotong Zeng, Qiuyang Jiang, Shanshan Zhang, Jin Wang, Qi Zhang, Feng Li, Dayong Xu

**Affiliations:** 1 Anhui Province Key Laboratory of Pollutant Sensitive Materials and Environmental Remediation, Huaibei Normal University, Huaibei, Anhui, China; 2 School of Life Sciences, Huaibei Normal University, Huaibei, Anhui, China; 3 Tolo Biotechnology Co., Ltd, Wuxi, Jiangsu, China; 4 Huaibei People’s Hospital, Huaibei, Anhui, China; Children's National Hospital, George Washington University, Washington, DC, USA

**Keywords:** *Mycoplasma pneumoniae*, CRISPR/Cas12a, ERA, one-pot detection, LFA

## Abstract

*Mycoplasma pneumoniae* (MP) is a significant pathogen associated with community-acquired pneumonia, with considerable infectious risks posed, particularly to children and immunocompromised individuals. The current methods for detecting MP in research and clinical settings are recognized as time-consuming, instrument-dependent, and prone to non-specific cross-reactivity. Therefore, the creation of a rapid and sensitive detection method is urgently required. In this study, the MP-ERA-Cas12a system, integrating enzyme restriction amplification (ERA) with clustered regularly interspaced short palindromic repeats (CRISPR)/Cas12a technology, was introduced. Three detection methods were evaluated: the two-pot system, a modified one-pot system, and a lateral flow assay (LFA) strip-based system. In the one-pot system, the amplification and detection steps were consolidated within a single reaction vessel, effectively minimizing the risk of contamination and false positives that may arise from the handling of multiple tubes. It was observed that the one-pot system generated a fluorescent signal within 1 h and produced 1.6 times higher fluorescence signal intensity compared to the two-pot system, achieving a detection limit of 1 copy/μL. In contrast, the LFA system facilitated rapid on-site screening, with visible band results appearing on the strip within 5 min of the reaction, and a detection limit of 10^2^ copies/μL was achieved. High specificity for MP was demonstrated by all methods. Significant advantages, including rapid processing, the absence of complex instrumentation, and ease of use are offered by this detection system, making it particularly suitable for resource-limited clinical settings. The system is seen as an efficient tool for the early diagnosis of MP, with substantial public health and clinical relevance.

**IMPORTANCE:**

This study successfully combined enzyme restriction amplification (ERA) with the specific detection capabilities of clustered regularly interspaced short palindromic repeats (CRISPR)/Cas12a. Based on the two-pot system established before, the one-pot system and lateral flow assay (LFA) system were developed for *Mycoplasma pneumoniae* (MP) detection. The MP-ERA-Cas12a system eliminates the need to open the lid during the reaction, reducing aerosol contamination, and minimizing the risk of false positives. The method does not require the use of advanced instruments or equipment and shows strong specificity while not being affected by other pathogens. As a new method of MP detection, the MP-ERA-Cas12a system has an important practical application prospect.

## INTRODUCTION

*Mycoplasma pneumoniae* (MP) was first identified by Eaton et al. in 1944 when it was isolated from the sputum of patients with atypical pneumonia (1). The organism appears as short, fine filaments, 2–5 µm in length. It resists Gram staining but takes on a pale purple coloration with Giemsa stain. MP infections originate from infected individuals or carriers, with the pathogen expelled through secretions from the nose, pharynx, throat, and trachea, spreading via droplets or aerosols (2). The infection is contagious during both the incubation and treatment periods. MP can cause a range of respiratory diseases and lead to various extrapulmonary complications (3). Preschool children and adolescents, whose immune systems are still developing, are particularly susceptible and represent the primary population affected by MP. Although MP infection is generally considered mild and self-limiting (4), its severity is often underestimated; over 10% of children hospitalized with MP require admission to intensive care (5). The clinical symptoms of MP infection are diverse and often mimic those of other respiratory pathogens, complicating diagnosis. Therefore, laboratory confirmation of MP is essential for effective clinical treatment. Diagnostic methods include culture, serological tests, antibody detection, and molecular biology techniques. While culture is considered the “gold standard” for diagnosing MP infection, culturing MP demands specialized culture media, and the growth of the culture may take 3 to 4 weeks or even longer (6). For optimal serological detection, at least two serum samples, collected 2 weeks apart, are typically required, making the procedure time-intensive. Additionally, the limited immune response observed in infants and elderly individuals (2), coupled with potential cross-reactivity with infections such as cytomegalovirus or Epstein-Barr virus (7), presents significant challenges to the sensitivity and specificity of MP detection. As a result, this method often yields a low detection rate, failing to meet the requirements for rapid clinical diagnosis. Antibody detection methods, including enzyme-linked immunosorbent assay, passive agglutination, and indirect immunofluorescence assays, are generally simple and fast but exhibit low sensitivity. These methods may also produce interference antibodies with high background levels in healthy individuals and non-specific cross-reactions, leading to false-positive results. Consequently, they are unsuitable for early screening of MP and are more appropriate for retrospective investigations (8–10) of MP infection. Molecular biology techniques, such as PCR technology and isothermal amplification, are the primary methods for detecting MP nucleic acid. However, many PCR-based detection methods are not well-suited for rapid detection or widespread use in resource-limited areas due to their complexity, long detection cycles, and dependence on specialized instruments and electricity (11–13).

Isothermal amplification is a novel class of molecular biotechnology that includes rapid detection methods such as loop-mediated isothermal amplification (LAMP) (14, 15) and recombinase polymerase amplification (RPA) (16, 17). These methods significantly outperform traditional nucleic acid detection technologies by reducing reaction times, eliminating the need for large equipment, and offering great potential for rapid diagnosis of MP. Enzymatic recombinase amplification (ERA), a patented technology, independently developed by GenDx Biotech Co., Ltd. in 2019, is an advanced version of RPA. This technique is similar to RPA and recombinase-aided amplification (RAA), except that the recombinant enzymes relied on in this reaction are derived from low-temperature phages and modify amino acids at specific sites (18). The modified DNA recombinase interacts with primers to form protein-DNA complexes, which locate homologous sequences in double-stranded DNA, initiating DNA synthesis and leading to exponential amplification of the target gene on the template (19). The high specificity of these enzymes minimizes the occurrence of mismatches, thereby reducing the likelihood of testing errors. ERA exhibits high detection sensitivity, allowing the amplification of trace nucleic acid templates to detectable levels. Specific genes can be amplified and qualitatively detected at constant temperatures of 35°C–42°C, with results observable using endpoint methods such as agarose gel electrophoresis. Typically, trace nucleic acid samples can be amplified to detectable levels within 15 min (20). The technology is characterized by its high stability and sensitivity, with a detection limit of 10^1^–10^2^ copies per reaction. The process is simple, requiring no specialized equipment and involving straightforward steps, making it accessible without professional skill training.

Clustered regularly interspaced short palindromic repeats (CRISPR) are specific DNA sequences found in prokaryotic organisms. CRISPRs, along with CRISPR-associated proteins (Cas proteins), play a crucial role in the adaptive immune systems of Archaea and bacteria, providing defense against invasive nucleic acids from foreign plasmids and phages (21, 22). In 2015, Zhang Feng et al. identified the V-type Cas12a (also known as Cpf1) within the CRISPR/Cas system in *Prevotella* and *Francisella* (23). This protein is a single RNA-guided CRISPR effector with DNA endonuclease activity, opening up possibilities for novel gene-editing tools. In April 2018, Doudna and her team discovered that the Cas12 protein exhibits a “trans-cleavage” effect, leading to the development of the DETECTR (DNA Endonuclease-Targeted CRISPR Transducer) system (24). Due to the challenges of PCR detection, which requires expensive equipment and trained technicians, there is an urgent need for a simpler amplification method that integrates CRISPR technology. Isothermal amplification does not rely on complex instruments and can be performed at a constant temperature using a simple heating device. The amplification products can be detected through agarose gel electrophoresis, real-time fluorescence, or lateral flow devices (LFD). Agarose gel electrophoresis can be used to analyze the size and quantity of amplicons, but it requires a longer reaction time and additional equipment. In contrast, fluorescent probes allow for real-time monitoring and analysis, while LFD is portable and easy to use, providing results that can be interpreted with the naked eye. Therefore, we chose to combine Cas12a detection with isothermal amplification technique. CRISPR detection techniques have been integrated with isothermal amplification. In 2024, the CRISPR/Cas system was utilized to detect high-priority STIs designated by the WHO (25), and in 2023, a portable system combining RPA and CRISPR/Cas system was developed for the visual detection of the monkeypox virus (26). The operating temperature of ERA (35°C–42°C) is similar to the optimal operating temperature of LbCas12a (37°C), so the combination of the two contributes to the development of a tube system. Cas12a can recognize and bind to target DNA with the guidance of crRNA. Upon forming a ternary complex with crRNA and the target sequence, the complex exhibits significant “trans-cleavage” activity. Utilizing this property of CRISPR/Cas12a, a system for pathogen detection has been developed.

In this research, we integrated the ERA isothermal amplification technique with the CRISPR/Cas12a system to develop and evaluate a novel rapid detection system for MP, referred to as the MP-ERA-Cas12a system. By utilizing the principle that once the target gene is recognized and cleaved by the CRISPR/Cas12a system, a “trans-cleavage” effect is triggered, which subsequently cleaves reporter probes, we introduced two types of reporter probes (fluorescent probes and strip probes) and established both the MP-ERA-Cas12a fluorescence and strip detection systems. Additionally, we developed a one-pot detection system that separates ERA amplification from CRISPR detection without requiring the tube to be opened for liquid transfers. This design allows the system to first amplify sufficient target sequences before proceeding with detection (27), enhancing sensitivity while simplifying the workflow and reducing the risk of false-positive results due to aerosol contamination. This ensures the accuracy of detection. Our goal is to create a detection system for MP that is rapid, cost-effective, highly sensitive, and specific.

## MATERIALS AND METHODS

### Materials and reagents

The recombinant plasmids, primers, and crDNAs used in this study were synthesized by Sangon Biotech (Shanghai, China). The sequences of the primers and crDNAs are detailed in Table 1. The Enzymatic Recombinant Isothermal Amplification Kit was purchased from GenDx Biotech Co., Ltd. (Suzhou, China). For the detection based on CRISPR/Cas12a, the following materials were sourced from Shanghai Tolo Port Biotechnology Co., Ltd. (Shanghai, China): the Cas12a High Yield crRNA Synthesis and Purification Kit, LbCas12a (Cpf1) Nuclease, 10× HOLMES Buffer, HOLMES ssDNA reporter (FAM), CRISPR-LFA ssDNA reporter, and the CRISPR single-system detection strip (FAM/FITC).

**TABLE 1:** Primers, crDNAs, and inserted fragments used in the study

Primer/crDNA	Sequence and modification (5′−3′)	Length (nt)
F1	AAGAAATCGGACTCGGAGGACAATGGTCAG	30
F2^*a*^	TGGGTGATACCGCTACCGTACCTCGCTTAC	30
F3	GGGGTTCTTCAGGCTCAGGTCAATCTGGC	29
R1	GTACAGAATCAGGATCGAGGCGGATCATTTGG	32
R2^*a*^	TGGCATCATCCTTACCGTTCTTGTCCTCT	29
R3	CGTCATTCATCTTTGCGGCGTTGCTTTCA	29
crDNA1	CGAACTGGAAAGGGCAGTACATCTACACTTAGTAGAAATTACTATAGTGAGTCGTATTA	59
crDNA2-1^*a*^	GCTTACTGTACGATGAACTTATCTACACTTAGTAGAAATTACTATAGTGAGTCGTATTA	59
crDNA2-2	AAGAGTTGCAAGTCTTCGCGATCTACACTTAGTAGAAATTACTATAGTGAGTCGTATTA	59
crDNA3-1	GCGTGGATCTCTCCCCCGTTATCTACACTTAGTAGAAATTACTATAGTGAGTCGTATTA	59
crDNA3-2	GTTACCAAGCACGAGTGACGATCTACACTTAGTAGAAATTACTATAGTGAGTCGTATTA	59
Fragment 1	AGAACGCCGAGGCGGACACCGCGAAGAGCAATGAAAAACTCCAGGGCGCTGAGGCCACTGGTTCTTCAACCACATCTGGATCTGGCCAATCCACCCAACGTGGGGGTTCGTCAGGGGACACCAAAGTCAAGGCTTTAAAAATAGAGGTGAAAAAGAAATCGGACTCGGAGGACAATGGTCAGCTGCAGTTAGAAAAAAATGATCTCGCCAACGCTCCCATTAAGCGGAGCGAGGAGTCGGGTCAGTCCGTCCAACTCAAGGCGGACGATTTTGGTACTGCCCTTTCCAGTTCGGGATCAGGCGGCAACTCCAATCCCGGTTCCCCCACCCCCTGAAGGCCGTGGCTTGCGACTGAGCAAATTCACAAGGACCTCCCCAAATGATCCGCCTCGATCCTGATTCTGTACGATGCGCCTTATGCGCGCAACCGTACCGCCATTGACCGCGTTGATCACTTGGATCCCAAGGCCATGACCGCGAACTATCCGCCCAGTTGAAGAACGCCCAAGTGAAACCACCACGGTTTGTGGGACTGAAAGGCGCGCGATGTTTTGCTCCAAACCACCGGGTTCTTCAACCCGCGCCGCCACCCCGAGTGGTT	601
Fragment 2^*a*^	CTGGTTTGAATATGTACCACGGATGGCAGTTGCTGGCGCTAAGTTCGTTGGTAGGGAACTCGTTTTAGCGGGTACCATTACCATGGGTGATACCGCTACCGTACCTCGCTTACTGTACGATGAACTTGAAAGCAACCTGAACTTAGTAGCGCAAGGCCAAGGTCTTTTACGCGAAGACTTGCAACTCTTCACACCCTACGGATGAGCCAATCGTCCGGATTTACCAATCGGGGCTTGAAGTAGTAGTAGTAGTAGTAGTCACAACGCACCCTACTACTTCCACAATAACCCCGATTGACAAGACCGTCCAATCCAAAATGTGGTTGATGCCTTTATTAAGCCCTGAGAGGACAAGAACGGTAAGGATGATGCCAAATACATCTACCCTTACCGTTACAGTGGCATGTGAGCTTGACAGGTATACAACTGGTCCAATAAGCTCACTGACCAACCAT	455
Fragment 3	AACCTGTTGGACCCCAACCAGGTTCGCACCAAGCTGCGCCAAAGCTTTGGTACAGACCATTCCACCCAGCCCCAGCCCCAATCGCTCAAAACAACGACACCGGTATTTGGGACGAGTAGTGGTAACCTCAGTAGTGTGCTTAGTGGTGGGGGTGCTGGAGGGGGTTCTTCAGGCTCAGGTCAATCTGGCGTGGATCTCTCCCCCGTTGAAAAAGTGAGTGGGTGGCTTGTGGGGCAGTTACCAAGCACGAGTGACGGAAACACCTCCTCCACCAACAACCTCGCGCCTAATACTAATACGGGGAATGATGTGGTGGGGGTTGGTCGACTTTCTGAAAGCAACGCCGCAAAGATGAATGACGATGTTGATGGTATTGTACGCACCCCACTCGCTGAACTGTTAGATGGGGAAGGACAAACAGCTGACACTGGTCCACAAAGCGTGAAGTTCAAGTCTCCTGA	461

^*a*^ Represents the primer/crDNA/fragment that was ultimately used.

### Bacterial strain and clinical samples

The strains used in this study are listed in Table 2. In this study, 112 clinical oropharynx swabs from Huaibei People’s Hospital were diagnosed by fluorescence quantitative PCR, including 34 positive samples and 78 negative samples. All the clinical samples were reviewed and approved by the Huaibei People’s Hospital Ethics Committee (no. 2024-052). Nucleic acids were extracted from these clinical samples using the GenePure 96 system.

**TABLE 2:** The strains used in this study

Strains	Abbreviation	No. of strains	MP-ERA-Cas12a^*a*^
*Mycoplasma pneumoniae*	MP	34	P
*Ureaplasma urealyticum*	UU	1	N
*Candida albicans*	CAL	1	N
*Candida tropicalis*	CTR	1	N
*Aspergillus fumigatus*	AF	1	N
*Escherichia coli*	*E. coli*	1	N
*Salmonella enteritidis*	SE	1	N
*Staphylococcus aureus*	SAU	1	N

^*a*^ P, positive; N, negative.

### Construction of recombinant plasmid

**Fig 1:**
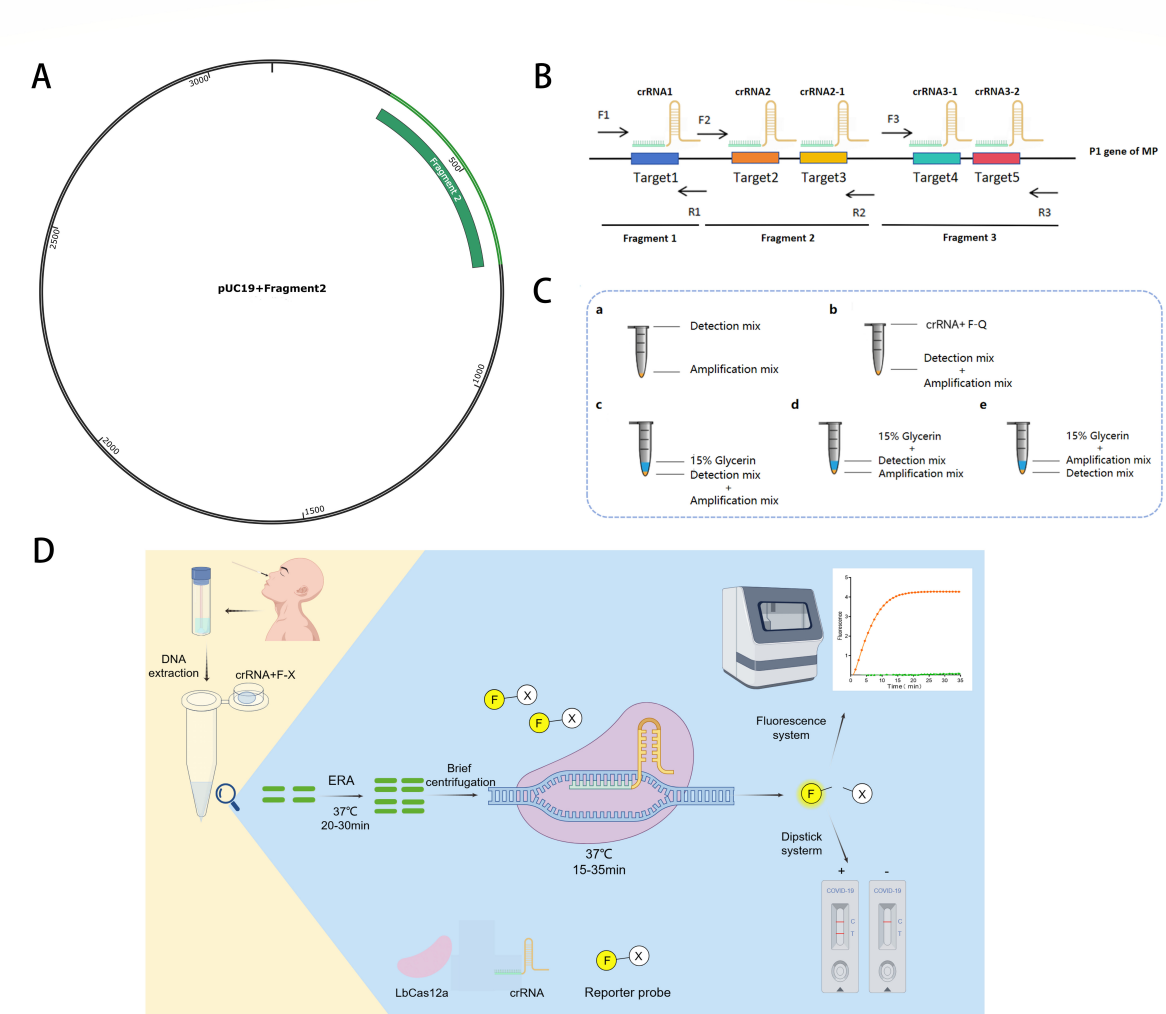
Design and workflow for the assay. (**A**) This image illustrates the positive recombinant plasmids designated for final use. (**B**) The schematic diagram of the MP-ERA-Cas12a system incorporates three targets, three pairs of primers, and five crRNAs. crRNA1 was employed to identify Target 1, amplified by primer F1R1. crRNA2-1 and crRNA2-2 identify Targets 2 and 3, amplified by primer F2R2. crRNA3-1 and crRNA3-2 identify Targets 4 and 5, amplified by primer F3R3. (**C**) After extracting DNA from the samples, the target sequence was amplified using the ERA technique over 20 to 30 min. Prior to amplification, crRNA and ssDNA were added to the tube cap. Upon completion of the ERA amplification, the LbCas12a-crRNA complex recognized the amplification products, triggering “trans-cleavage” activity that cleaved the ssDNA reporter within approximately 35 min. Subsequent analyses can be conducted by observing fluorescence signals or using lateral flow assay (LFA) test strips. (**D**) Different sampling methods were tested when setting up the MP-ERA-Cas12a one-pot system: (a) the detection mixture was added to the cap, and the amplification mixture was added to the tube base. (**b**) crRNA and F-X were added to the cap, while the detection and amplification mixtures were added to the base. (**c**) Both detection and amplification mixtures were added to the base of the tube. (**d**) The amplification mixture was added to the base and separated from the detection mixture by 15% glycerin. (**e**) The detection mixture was added to the base, with 15% glycerin separating it from the amplification mixture. F-X: F-Q or F-B, F-Q was used in the fluorescence detection system, F-B was used in LFA detection system. T, test line; C, control line.

Based on the sequences of the MP P1 gene, three unique sequences were identified and subsequently cloned into the pUC19 vector to construct the recombinant plasmid. A structural diagram of the final recombinant plasmid is presented in Fig. 1A. The nucleic acid concentration of the synthesized plasmids was measured using an ultraviolet spectrophotometer. The plasmid concentration was converted to copy number using NEBioCalculator version 1.15.8. The DNA sample was then re-suspended in ddH_2_O and stored at −20°C for future use.

### Design of isothermal amplification primers and crDNA

According to the Primer Design Principles of ERA, primers were designed using Primer Premier 5.0 software. The specificity of these primers was evaluated through BLAST analysis. The target location was identified using the PAM site (TTTV) of Cas12a and CRISPR RGEN Tools. The final crRNA sequence should be 5′-AAUUUCUACUAAGUGUAGAUNNNNNNNNNNNNNNNNNNNN-3′, with the underlined portion being the specific target sequence complementary to the spacer region. The locations of the ERA primer and crRNA are depicted in Fig. 1B, while the schematic diagram of MP-ERA-Cas12a is presented in Fig. 1C. In this study, the MP P1 gene fragments were used to design clustered regularly interspaced short palindromic repeat DNA (CRISPR DNA, crDNA). crDNAs were then transcribed into crRNAs using the Cas12a High Yield crRNA Synthesis and Purification Kit.

### Screen the best primer-crRNA combination

At the outset of the study, the most effective combination of components was screened. The optimal composition can vary across different experimental systems, making it crucial to explore and determine the most suitable combination before proceeding with the subsequent experimental schemes. Throughout the experimentation process, maintaining uniform environmental conditions and consistent quantities for each combination is essential to ensure that any differences in results are solely due to the varying efficacy of the combinations. Different primer pairs were designed for distinct target sequences, and upstream and downstream candidate primers (F1, F2, F3, R1, R2, R3) were synthesized and paired to form F1R1, F2R2, and F3R3 primer pairs. Considering the varying cutting efficiencies of different crRNA corresponding to different target sequences, five crRNA with high predicted cutting efficiencies were synthesized. To select the optimal combination, primers and crRNA were introduced into the MP-ERA-Cas12a fluorescence reaction system for detection. Combine the primer pair F1R1 with crRNA1 (F1R1-crRNA1), the primer pair F2R2 with crRNA2-1 (F2R2-crRNA2-1), the primer pair F2R2 with crRNA2-2 (F2R2-crRNA2-2), the primer pair F3R3 with crRNA3-1 (F3R3-crRNA3-1), and the primer pair F3R3 with crRNA3-2 (F3R3-crRNA3-2). The template concentration was set at 10^7^ copies/μL, with ddH_2_O serving as the negative control. Each sample was repeated three times. The optimal primers were identified by analyzing the fluorescence production time and the patterns in the fluorescence curves.

### Enzymatic recombinant isothermal amplification

ERA amplification was performed using the Enzymatic Recombinant Isothermal Amplification Kit. The total ERA reaction system consisted of 50 µL, including 20 µL of solvent, 2.5 µL each of 10 µM forward and reverse primers, and 10^7^ copies of the plasmid template. The remaining volume was made up with ddH_2_O to bring the total to 48 µL. The mixture was thoroughly mixed and transferred to the basic amplification reagent provided in the kit. Subsequently, 2 µL of activator was added to the tube cap. The activator was then incorporated into the premix through short centrifugation at room temperature, followed by brief oscillation and mixing, and a final rapid centrifugation. The reaction was then incubated at 37°C for 20 min, with the expansion temperature maintained using a water bath. After the reaction, the amplified products were purified for subsequent experiments.

### Establishment and optimization of the MP-ERA-Cas12a two-pot system

LbCas12a was utilized for trans-cleavage detection in this research. When establishing the MP detection system in conjunction with Cas12a, system optimization was performed. This was achieved by varying the concentration of the F-Q reporter and the ratio of Cas12a to crRNA (Cas12a:crRNA). The positive recombinant plasmid containing 10^7^ copies was used as the template. Concentrations of the F-Q reporter were tested at 100 nM, 200 nM, 300 nM, 400 nM, and 500 nM, while Cas12a:crRNA ratios of 2:1, 1.5:1, 1:1, 1:1.5, 1:2, 1:3, and 1:4 were evaluated. The specific concentration or volume of each component in the system is shown in Table 3. The optimal ratio of the components was ultimately selected based on the fluorescence values obtained from the results.

**TABLE 3:** The corresponding system of three detection methods^*a*^

	Component	Two-pot system	One-pot system	LFA system
A	ERA solvent	20 µL	4 µL	4 µL
	F	500 nM	250 nM	250 nM
	R	500 nM	250 nM	250 nM
	Plasmid template	10^7^ copies	10^7^ copies	10^7^ copies
	ERA activator	2 µL	2 µL	2 µL
	DEPC water	Up to 50 µL		
		Take 1 µL A product into B		
B	F-X^*b*^	300 nM	300 nM	40 nM
	crRNA	625 nM	312.5 nM	312.5 nM
	10× Holmes buffer	2 µL	1 µL	1 µL
	LbCas12a	250 nM	125 nM	125 nM
	DEPC water	Up to 20 µL	Up to 20 µL	Up to 20 µL

^*a*^ A: ERA system, B: Cas12a system.

^*b*^ F-X stands for F-Q in the two-pot and one-pot systems, and for F-B in the LFA systems.

### Establishment and optimization of MP-ERA-Cas12a one-pot system

The MP-ERA-Cas12a system was transformed into a one-pot reaction system, capable of performing isothermal amplification and detection in the same tube. Five sampling methods were explored, as depicted in Fig. 1D. This one-pot system requires three premixes: the ERA amplification mix, the Cas12a detection mix, and the reaction starter mix. The reaction starter mix is added to the cap of the reaction tube. The concentrations of the components (Table 3) in these premixes have been optimized. The ERA mixture includes 4 µL of ERA solvent, 6.85 µL of ddH_2_O, 10^7^ copies of a plasmid template, 0.5 µL each of 10 µM forward and reverse primers, 2 µL of ERA activator, and one-fifth of a tubular lyophilized reagent. The Cas12a mixture contains 0.25 µL of 10 µM LbCas12a and 1 µL of 10× HOLMES buffer. The reaction starter mixture is composed of 1.5 µL of 4 µM crRNA and 2 µL of 3 µM F-Q reporter. Before the reaction, 15.25 µL of the ERA mixture and 1.25 µL of the Cas12a mixture are added to the base of the tube, while 3.5 µL of the reaction starter mixture is placed in the lid of the tube. The system is then incubated at 37°C for 20 min to promote amplification. Afterward, the reaction starter mixture falls to the base through a brief centrifugation. Finally, the reaction is conducted in the LightCycler 96 Instrument at 37°C for 35 min.

### Establishment and optimization of MP-ERA-Cas12a LFA system

In this study, the trans-cleavage function of the LbCas12a protein was integrated with ERA isothermal amplification technology. A biotinylated ssDNA (single-stranded DNA) reporter probe (F-B) was introduced to enable direct observation of test strip results with the naked eye. In the establishment of the lateral flow assay (LFA) system, the ERA amplification and Cas12a detection systems remained consistent, with the only modification being the replacement of the reporter probe (F-Q) with a FAM and biotin-modified reporter probe (F-B).

The F-B probe functions similarly to an antigen in LFA. Both excessively high and low antigen concentrations can result in a “hook effect.” Therefore, it is essential to investigate the optimal concentration of the probe in this study (Table 3). This involves creating a series of dilution gradients by taking varying volumes of the probe, which was initially dissolved in water or a dilution solution. These dilutions (10 µM, 1 µM, 100 nM, 10 nM, and 1 nM) were then inserted into an empty test strip, and the resulting bands were observed. The optimal condition is defined as the lowest concentration of the probe at which the test line (T-line) is not visible.

While the fluorescence system can provide preliminary results in under 10 min, the test strips used in this study have a slight incidence of false positives and notable differences compared to the fluorescence system. To minimize any confusion caused by these discrepancies, the incubation time for the MP-ERA-Cas12a LFA system was systematically optimized. Reaction tubes were maintained at 37°C for different durations (5, 10, 15, 20, 30, and 60 min). After incubation, the reaction product was transferred to the sample absorption pad of the test strip. The template concentration was 10^7^ copies/µL, with deionized water (ddH_2_O) serving as the negative control. Each sample underwent three repetitions. Once completed, the amplified product and test strips were placed in a sealed bag for proper disposal.

### Sensitivity and specificity of the MP-ERA-Cas12a system

To evaluate the sensitivity of the three methods, we examined a range of nucleic acid concentrations. For each reaction, the MP nucleic acid was serially diluted from 10^7^ copies to 1 copy, with a dilution factor of 10 for sensitivity analysis. To assess the specificity of the MP-ERA-Cas12a system, we used MP and six non-MP strains, with distilled water serving as a blank control. Nucleic acids were extracted from all bacterial samples using a Rapid Bacterial Genomic DNA Isolation Kit, while fungal nucleic acids were obtained through the Bead Beating method. In both analyses, the MP-ERA-Cas12a system was conducted according to the previously mentioned three methods, with each system repeated three times for consistency.

### Clinical feasibility confirmation of the MP-ERA-Cas12a system

In order to verify the clinical feasibility of the MP-ERA-Cas12a system, we collected 112 clinical samples in Huaibei People’s Hospital, of which 34 were positive and 78 were negative. DNA was extracted from clinical samples using the GenePure 96 system. The extracted DNA was then evaluated using the MP-ERA-Cas12a system.

## RESULTS

### Principle of MP-ERA-Cas12a detection system

The schematic based on MP-ERA-Cas12a is illustrated in Fig. 1C.

First, in the ERA system, DNA recombinases bind to primers to form protein-DNA complexes, which recognize homologous sequences in MP double-stranded DNA and initiate DNA synthesis, leading to exponential amplification of target genes. In the Cas12a detection system, under the guidance of crRNA, Cas12a recognizes the target sequence through the PAM site and cuts it, and the trans-cleavage activity of Cas12a is activated at the same time, randomly cutting ssDNA, that is, the reporting probes.

In the one-pot experiment, the amplification system activated at 37°C, resulting in target amplification through the ERA system. Due to the absence of crRNA, Cas12a activity could not be initiated. After the ERA reaction, crRNA and the F-Q reporter were added, and the system was briefly centrifuged to initiate trans-cleavage detection activities. Cas12a activates its cis-cleavage and trans-cleavage functions by pairing with crRNA to recognize and bind amplified products. Notably, the 3.5 µL Cas12a reaction starter mixture remained on the cap due to surface tension, and the one-pot system minimizes aerosol contamination risks by eliminating the need to open the cap. This approach enables specific detection guided by crRNA, reducing false positives from non-specific amplification while facilitating signal amplification and detection. Consequently, this platform serves as a simple and sensitive tool for nucleic acid detection.

In the MP-ERA-Cas12a LFA system, the reporter probe is labeled with biotin at one end and fluorescein (FAM) at the other. The lateral chromatographic test strip features two lines: the lower control line (C line) and the upper detection line (T line). Avidin coats the C line, while a sheep anti-mouse secondary antibody coats the T line. The anti-FAM monoclonal antibody is labeled with colloidal gold. The complete CRISPR probe captures all colloidal gold at the C line. When the probe is cleaved by the Cas enzyme, the bound colloidal gold fragment no longer captures at the C line, forming the T line instead. The presence or absence of the T line indicates Cas enzyme activation: visible T lines denote positive results, while invisible T lines indicate negatives. The presence of the C line confirms proper strip function. Compared to qPCR methods that require large instruments, this approach is more convenient, effectively meeting rapid detection needs in primary healthcare settings.

### Screen the best primer-crRNA combination

In this experiment, three recombinant plasmids, three pairs of amplification primers, and five crRNAs were designed, as shown in Fig. 1B. Results (Fig. 3) indicated that after adding the combined primer pairs and crRNAs to the system, fluorescence curves from all five groups exhibited linear increases. The control group using ddH_2_O produced increased fluorescence signals in F1R1-crRNA1, F3R3-crRNA3-1, and F3R3-crRNA3-2, suggesting these combinations lack specificity for MP detection; therefore, their results did not support MP testing (Fig. 3A, D, and E).

For F2R2-crRNA2-1 and F2R2-crRNA2-2, the fluorescence curve for F2R2-crRNA2-1 showed a steep increase, reaching saturation within 20 min. In contrast, F2R2-crRNA2-2 exhibited a gentler slope and did not reach saturation after 35 min. Additionally, the fluorescence value of F2R2-crRNA2-1 at 35 min was 1.3 times greater than that of F2R2-crRNA2-2 and approximately 35 times that of the negative control (Fig. 3B and C). Consequently, F2R2-crRNA2-1 was selected for its higher fluorescence intensity.

**Fig 2:**
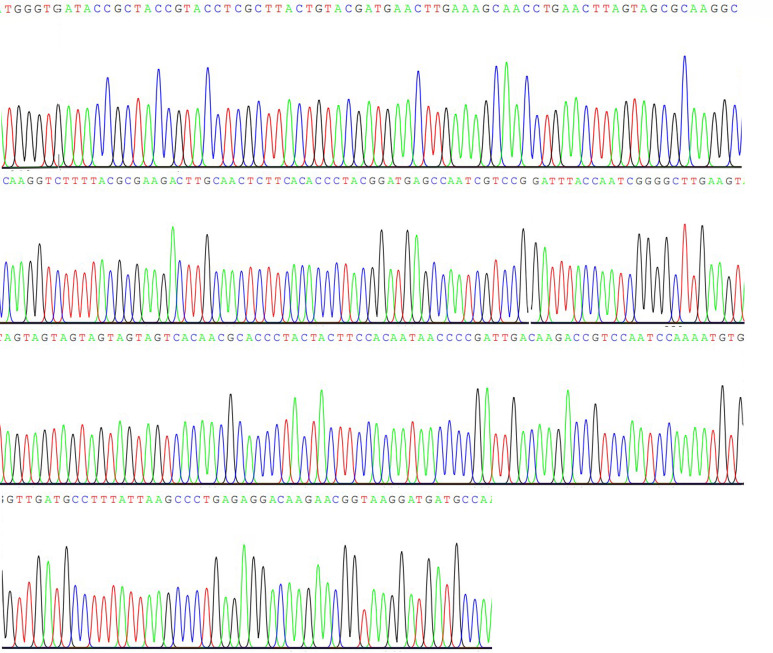
DNA sequencing verified the target sequence amplified by F2R2.

The combination of forward primer 2 and reverse primer 2 (F2R2), which reached the reaction plateau, was identified as the most effective primer. DNA sequencing was used to verify the target sequences amplified by F2R2 (Fig. 2). Results also indicate that crRNA2-1 serves as an ideal guide RNA for targeting the P1 gene. Therefore, the complementary DNA sequence of crRNA1 was determined to be the most suitable target sequence (recombinant plasmid 2).

### Establishment and optimization of the MP-ERA-Cas12a two-pot system

**Fig 3:**
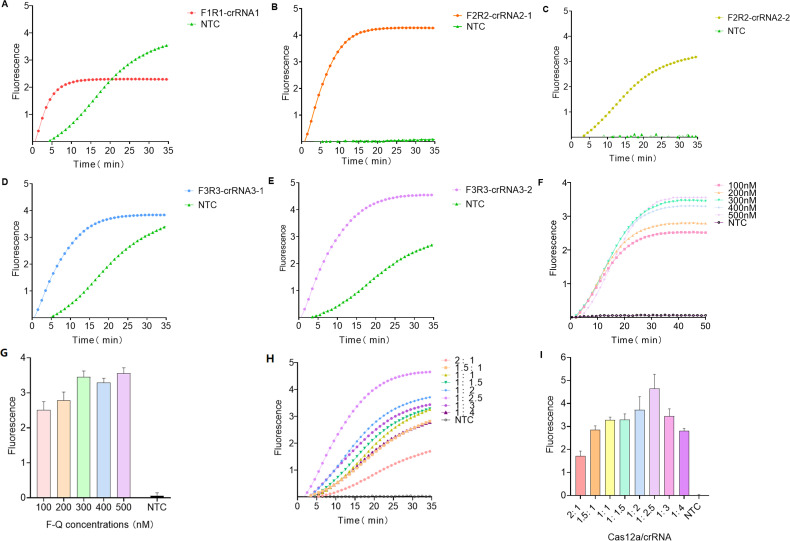
Fluorescence curves produced by different reactions. (**A–E**) Fluorescence curves generated by each combination detecting positive samples. (**F**) Fluorescence curves were produced using different concentrations of F-Q. (**G**) Fluorescence values were obtained with varying concentrations of F-Q at 40 min. (**H**) Fluorescence curves produced with different proportions of Cas12a:crRNA. (**I**) Fluorescence values were obtained with varying proportions of Cas12a:crRNA at 35 min. NTC, no template control.

To optimize the system, different concentrations of F-Q probes were introduced. As shown in Fig. 3F, the fluorescence curve plateaued after approximately 30 min at F-Q concentrations of 100 and 200 nM. At concentrations of 300, 400, and 500 nM, the curve plateaued after about 35 min. Detected fluorescence intensity was significantly higher than that of negative controls. Notably, at plateau, fluorescence intensity of F-Q probes at concentrations ranging from 300 to 500 nM exceeded that of lower concentrations (Fig. 3G). Since no significant differences in fluorescence values were observed among 300, 400, and 500 nM, 300 nM F-Q probes were selected as the optimal condition for the MP-ERA-Cas12a fluorescence system to conserve materials.

The Cas12a to crRNA ratio significantly affects the trans-cleavage performance of the CRISPR/Cas12a system. With a Cas12a:crRNA ratio of 1:2.5, the fluorescence value (4.651 ± 0.616) of the reaction system exceeded that of other ratios, and the fluorescence curve exhibited a steep upward linear trend, plateauing after 35 min (Fig. 3H and I). Thus, the optimal Cas12a:crRNA ratio was determined to be 1:2.5.

### Establishment and optimization of MP-ERA-Cas12a one-pot system

**Fig 4:**
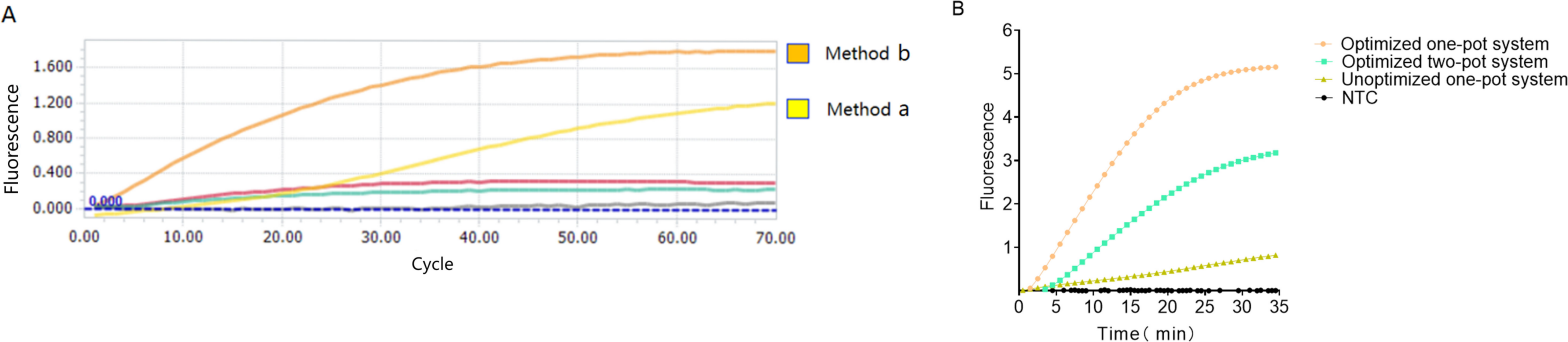
The fluorescence curves produced by five sampling methods. (**A**) Unlabeled curves correspond to the fluorescence data from methods c, d, and e. (**B**) Comparison of the one-pot system’s effect after optimization with other methods. NTC, no template control.

During one-pot system development, five sampling methods were evaluated. Results showed that three reactions with 15% glycerol (Fig. 1D) exhibited no significant fluorescence. In contrast, reactions with only the activator (crRNA and F-Q) added to the cap displayed higher fluorescence than those with the entire detection system added (Fig. 4A). Therefore, the next optimization step will follow the sample addition method illustrated in Fig. 1D.

As shown in Fig. 4B, the fluorescence value of the unoptimized one-pot system is relatively low (0.826241741 ± 0.040), while the optimized fluorescence value is approximately 6.24 times greater. This underscores the importance of system optimization in establishing the one-pot system. Comparing the optimized one-pot system to the commonly used two-pot system, the one-pot system reaches its plateau earlier and exhibits fluorescence intensity 1.6 times higher than the two-pot system, given the same template DNA concentration. This approach not only streamlines operational steps but also enhances fluorescence output.

### Establishment and optimization of the MP-ERA-Cas12a LFA system

**Fig 5:**
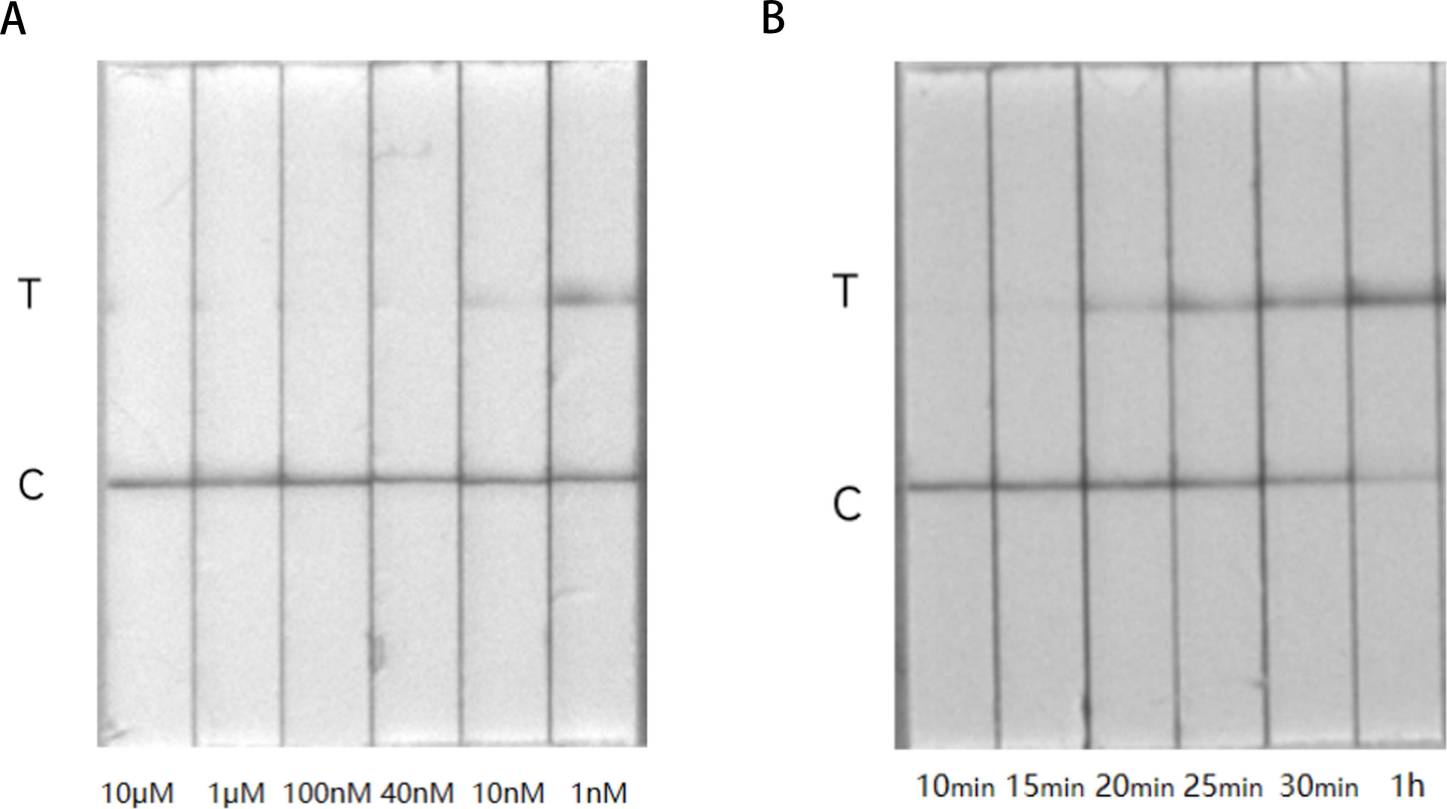
The optimized MP-ERA-Cas12a LFA system. (**A**) LFA system that adds different concentrations of F-B. (**B**) LFA system incubated at different times.

To minimize the T-line strength, we determined the optimal F-B probe concentration for the MP-ERA-Cas12a LFA system. The negative control showed bands only on the T-line, while decreasing F-B probe concentrations led to increased intensity of the T-line band and a lighter C-line band. Notably, at an F-B concentration of 40 nM, the T-line intensity matched that at 100 nM, significantly lower than the C-line intensity (Fig. 5A). Therefore, 40 nM is recommended as the optimal probe concentration for the MP-ERA-Cas12a LFA system.

The MP-ERA-Cas12a LFA system serves as the endpoint monitoring system, making incubation time critical for results. As shown in Fig. 5B, increasing incubation time from 20 min correlated with higher T-line intensity and lower C-line intensity. A noticeable color difference between positive and negative results was evident after 25 min. Consequently, we selected 25 min as the optimal incubation time for the MP-ERA-Cas12a test strip system due to the more pronounced color difference and shorter duration.

### Sensitivity and specificity of the MP-ERA-Cas12a system

**Fig 6:**
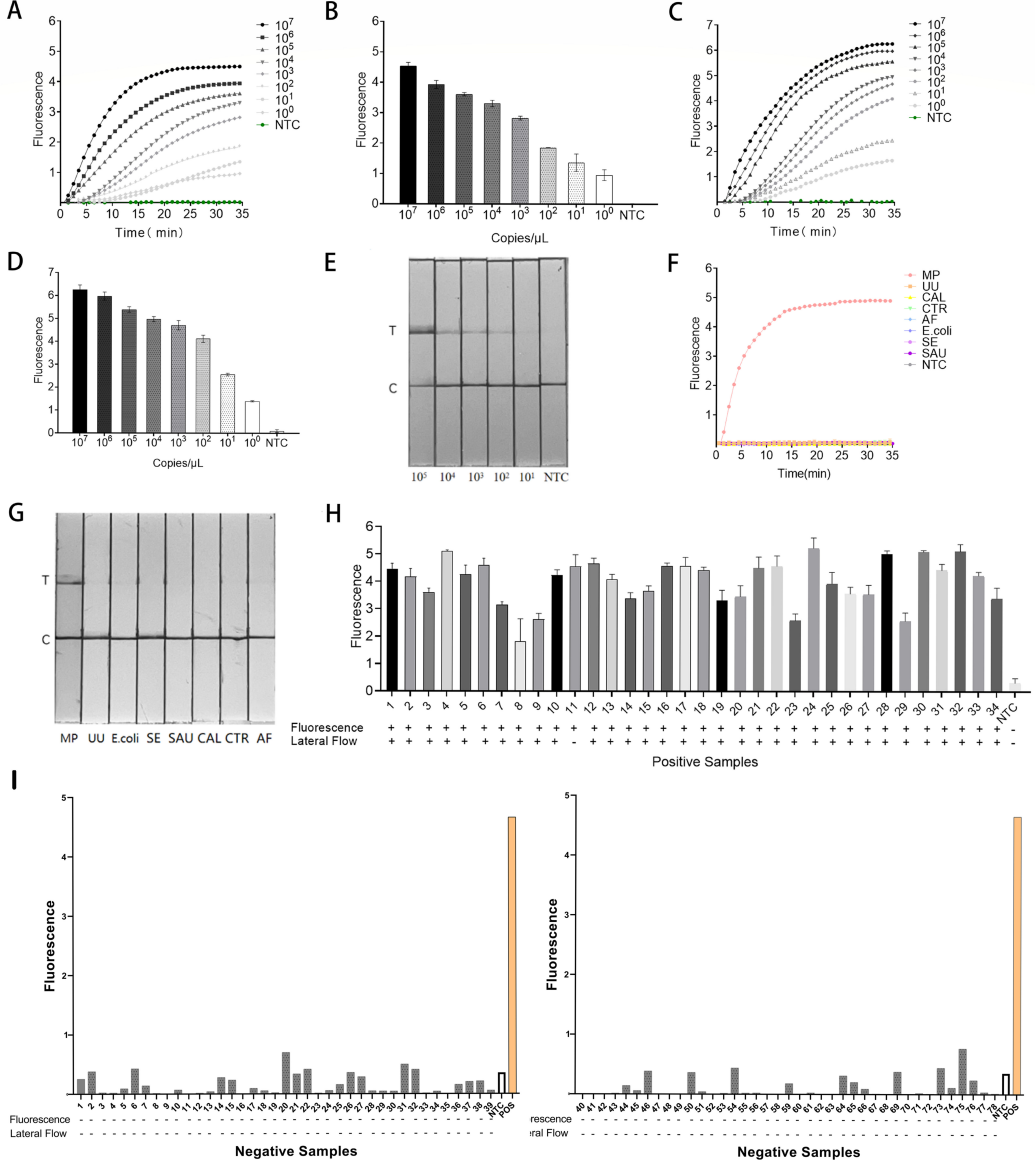
Sensitivity and specificity analysis of the MP-ERA-Cas12a system. (**A**) The fluorescence curves produced by the two-pot system using different template copies. (**B**) The fluorescence curves from the two-pot system at 35 min with varying template copies. (**C**) Fluorescence curves generated by the one-pot system with different template copies. (**D**) Fluorescence curves from the one-pot system at 35 min with varying template copies. (**E**) Results produced on the LFA system when using different copies of the template in a one-pot system. (**F**) Specificity analysis of the MP-ERA-Cas12a fluorescence system, testing against various pathogens: MP, *Mycoplasma pneumoniae*; UU, *Ureaplasma urealyticum*; CAL, *Candida albicans*; CTR, *Candida tropicalis*; AF, *Aspergillus fumigatus*; *E. coli*, *Escherichia coli*; SE, *Salmonella enteritidis*; SAU, *Staphylococcus aureus*. (**G**) Specificity analysis of the MP-ERA-Cas12a LFA system, testing against various pathogens. (H) The MP-ERA-Cas12a system was used to validate 34 known positive samples. (**I**) The MP-ERA-Cas12a system was used to validate 78 known negative samples. NTC, no template control; +, positive; −, negative.

To assess the sensitivity of the MP-ERA-Cas12a system, we evaluated various nucleic acid concentrations (Fig. 6). We systematically reduced the template concentration from 10^7^ to 1 copy/µL, resulting in progressively decreasing fluorescence intensity measured at 35 min, as presented in Fig. 6B and D. For both two-pot and one-pot methods, the detection limit reached as low as 1 copy/µL. A significant difference in fluorescence intensity appeared within 20 min following Cas12a activation, demonstrating the system’s high sensitivity for MP. Higher copy numbers (10^7^, 10^6^, and 10^5^) exhibited earlier plateauing fluorescence curves compared to lower copy numbers (Fig. 6A and C). Additionally, the one-pot system yielded fluorescence values approximately 1.5 times greater than the two-pot system. The LFA system also detected varying template copies (Fig. 6E). At a copy number of 10^1^, the T-line band was weak, but a strong band appeared with copy numbers exceeding 10^1^ copies/µL, establishing the LFA’s actual detection limit at 10^2^ copies/µL.

Specificity is crucial for detection systems. We validated the MP-ERA-Cas12a system’s specificity using nucleic acids from various pathogens, including UU, *E. coli*, SE, SAU, CAL, CTR, and AF. As shown in Fig. 6F, only MP exhibited significant fluorescence, while other pathogens did not. In the LFA system for MP detection (Fig. 6G), only MP displayed prominent T-line bands, confirming the MP-ERA-Cas12a system’s strong specificity for MP.

### Clinical feasibility confirmation of the MP-ERA-Cas12a system

This study evaluated the MP-ERA-Cas12a fluorescence detection system and LFA system using 34 known positive samples and 78 known negative samples. The fluorescence detection system successfully detected all 34 positive samples, while the test strip identified 33 (Fig. 6H). The results of 78 negative samples were consistent with those of fluorescence quantitative PCR (Fig. 6I). Using fluorescence results as the gold standard, the positive predictive rate of the MP-ERA-Cas12a LFA system was calculated at 97.06%, and the negative predictive rate was calculated at 100%, indicating high concordance between the two methods in detecting MP. This finding further substantiates the reliability and effectiveness of the MP-ERA-Cas12a system for clinical sample detection, underscoring its strong clinical applicability.

## DISCUSSION

In 2017, Doudna’s team (28) discovered that the CRISPR system targets double-stranded DNA for cleavage while activating the non-specific endonuclease activity of Cas12a, leading to indiscriminate cleavage of single-stranded DNA. This finding paved the way for *in vitro* nucleic acid detection. In 2018, Doudna’s team (22) integrated RPA isothermal amplification with CRISPR/Cas12a to develop the DETECTR system, enabling rapid, straightforward, and real-time detection of min DNA quantities in samples.

MP, a common respiratory pathogen, can cause pharyngitis, tracheitis, and bronchitis. While most infections are self-limiting, some may progress to pneumonia. Numerous methods exist for detecting MP. However, these typically require specialized procedures and advanced laboratory equipment, making them unsuitable for field-based testing due to high costs. Zhongliang Deng et al. (29) integrated ERA amplification technology, the CRISPR/Cas12a system, and lateral flow assay to develop the LFA system. This system enables direct visual observation of test strip results without instruments, simplifying analysis. The CRISPR component mitigates non-specific signals from untargeted ERA amplification, while ERA enhances CRISPR system sensitivity. Both components operate at 37°C, simplifying procedural steps. However, if amplification and CRISPR detection reagents are premixed prior to the reaction, Cas12a can continuously digest amplification products and primers at low template concentrations, reducing amplification efficiency (30, 31). To address this, most approaches separate amplification and detection reactions using the widely employed two-pot system. However, manual transfer of amplification products complicates the process and increases contamination risk (32). Many studies have adopted a “physical isolation” strategy to resolve this issue (33), though these solutions can render the operational process cumbersome.

Most methods that combine ERA amplification with CRISPR/Cas detection require a step-by-step operation. ERA amplification is performed first, and then CRISPR/Cas detection-related reagents are added to the system for detection. This is easy to produce aerosol contamination, resulting in incorrect detection results, and it has high requirements for the testing environment. Therefore, we hope to develop a fast and sensitive detection method without pipetting operation to detect MP and provide ideas and technical support for field detection. The introduction of the one-tube method allows us to directly mix ERA and CRISPR/Cas12a reagents in the same tube for reaction, ensuring that ERA amplification and CRISPR detection are isolated without opening the cap for pipetting. This ensures the system can first amplify enough target sequences and then conduct detection, improving sensitivity at the same time. The operation process is reduced, and the possibility of aerosol contamination is greatly reduced (26).

Despite the high specificity and sensitivity of CRISPR technology in diagnostics, it has limitations. Precise conditions such as temperature control and target nucleic acid pre-amplification are required, adding to complexity and time. High reagent costs and technical demands for sample preparation and reaction control impede its application in low-resource areas. False positives due to off-target effects also undermine its reliability. These issues need resolution to realize their potential (34).

In this study, we established a more sensitive one-pot system that combines ERA amplification with Cas12a detection, yielding higher fluorescence values than the traditional two-pot system. This approach facilitates rapid detection of MP in a single tube, eliminating the need for additional primer or crRNA design, as well as physical isolation methods. The reaction activator, consisting of crRNA and F-Q, was simply added to the tube lid, while the remaining components were mixed at the base. This method effectively addresses contamination issues arising during the secondary uncapping. The MP-ERA-Cas12a system demonstrated high sensitivity, achieving a low limit of detection of 1 copy/µL for MP. Compared with other rapid detection methods, such as LAMP (35) and RAA (36), the ERA/CRISPR-Cas12a fluorescence system showed higher sensitivity. When paired with an LFA strip, sensitivity can reach 10^2^ copies/µL. This system has better sensitivity, and a lower load of MP can be detected. This system overcomes the limitations of large instruments and enhances testing accessibility in remote areas. Furthermore, the MP-ERA-Cas12a system proved effective in clinical sample testing.

Employing the system established in this study, we analyzed 34 positive and 78 negative clinical samples from hospitals. All of these 112 samples had been diagnosed using fluorescent quantitative polymerase chain reaction (qPCR). Results from the LFA were consistent with those obtained from qPCR, indicating strong agreement between the two methods. The final positive detection rate obtained from the positive samples was 97.06%, and the positive detection percentage obtained from the negative samples was 0%. And the negative prediction rate was 100%. Compared with other CRISPR-based side-flow chromatography techniques, the positive predictive value and negative predictive value of the ERASE system were 90.67% and 99.21%, respectively (37). Compared to the CRISPR/Cas12a MP detection system developed in 2022, which has a positive prediction rate of 92.86% (29), this system has a relatively high prediction rate. Additionally, detection of other pathogens using this system yielded negative results, demonstrating high specificity. However, limitations include the selection of only eight pathogens for specificity evaluation, excluding MP, which may not provide a comprehensive assessment. Future experiments will include additional pathogens associated with respiratory tract infections to further investigate the specificity of this detection system.

In conclusion, this study successfully integrated ERA with the specific detection capabilities of CRISPR/Cas12a to develop a one-pot and LFA system for MP detection, enhancing the previously established two-pot system. The MP-ERA-Cas12a system eliminates the need to open the lid during reactions, reducing aerosol contamination and minimizing the risk of false positives. Additionally, results are produced within 1 h. The one-pot system achieved a detection sensitivity of 1 copy/μL, while the LFA demonstrated a sensitivity of 10^2^ copies/μL, without requiring advanced instruments or equipment. This system exhibits robust specificity, unaffected by other pathogens. In terms of the economic aspect, compared with the traditional culture-based methods for detecting MP, the CRISPR/Cas12a-ERA isothermal amplification method in this study is more efficient. Since it doesn't require long-term culturing, this significantly reduces labor costs. Moreover, while some complex molecular diagnostic techniques require expensive equipment, our method can be implemented with relatively simple and inexpensive instruments, thus reducing the overall detection cost. It is highly convenient for detecting MP in remote mountainous areas (38). The MP-ERA-Cas12a technique shows significant promise as a novel approach for MP detection, with meaningful practical applications.
